# 4,4′-[(2R*,3R*,4R*,5R*)-3,4-Dimethyl­tetra­hydro­furan-2,5-di­yl]diphenol

**DOI:** 10.1107/S1600536812039359

**Published:** 2012-09-26

**Authors:** Juan Manuel de Jesús Favela-Hernández, María del Rayo Camacho-Corona, Sylvain Bernès, Marcos Flores-Alamo

**Affiliations:** a Facultad de Ciencias Químicas, Universidad Autónoma de Nuevo León, UANL, Avenida Universidad S/N, Ciudad Universitaria, San Nicolás de los Garza, Nuevo León CP 66451, Mexico; b Facultad de Química, Universidad Nacional Autónoma de México, México DF 04510, Mexico

## Abstract

The title mol­ecule, C_18_H_20_O_3_, is a furan­oid lignan extracted from the leaves of *Larrea tridentata*. The relative absolute configuration for the four chiral centers was established, showing that this compound is 4-*epi*-larreatricin, which has been previously reported in the literature. The mol­ecule displays noncrystallographic *C*_2_ symmetry, with the methyl and phenol substituents alternating above and below the mean plane of the furan ring. The conformation of this ring is described by the pseudorotation phase angle *P* = 171.3° and the maximum out-of-plane pucker ν_m_ = 37.7°. These parameters indicate that the furan ring adopts the same conformation as the ribose residues in B-DNA. The packing is dominated by inter­molecular O—H⋯O hydrogen bonds. The phenol hy­droxy groups form chains in the [110] direction and these chains inter­act *via* O—H⋯O(furan) contacts.

## Related literature

For the extraction, synthesis, characterization and biological activity of the title compound, see: Konno *et al.* (1990 ▶); Moinuddin *et al.* (2003 ▶); Favela-Hernández *et al.* (2012 ▶). For the conformational analysis of sugar rings, see: Altona & Sundaralingam (1972 ▶); Sun *et al.* (2004 ▶). For an example of another naturally occurring furan­oid lignan, see: Soepadamo *et al.* (1991 ▶).
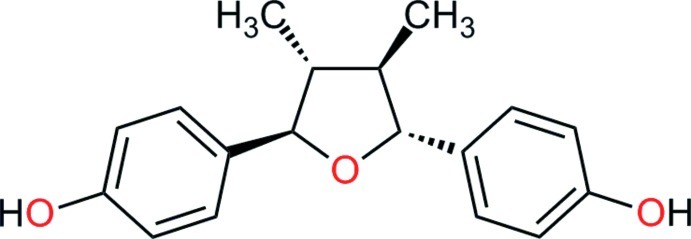


## Experimental

### 

#### Crystal data

C_18_H_20_O_3_
*M* *_r_* = 284.34
Monoclinic, 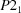

*a* = 6.4225 (4) Å
*b* = 12.4973 (7) Å
*c* = 9.8176 (7) Å
β = 101.243 (6)°
*V* = 772.88 (9) Å^3^
*Z* = 2
Mo *K*α radiation
μ = 0.08 mm^−1^
*T* = 298 K
0.36 × 0.26 × 0.21 mm

#### Data collection

Agilent Xcalibur (Atlas, Gemini) diffractometer
Absorption correction: analytical [*CrysAlis PRO* (Oxford Diffraction, 2009 ▶), based on expressions derived by Clark & Reid (1995 ▶)] *T*_min_ = 0.980, *T*_max_ = 0.985
5223 measured reflections
1592 independent reflections
1107 reflections with *I* > 2σ(*I*)
*R*_int_ = 0.041

#### Refinement

*R*[*F* ^2^ > 2σ(*F* ^2^)] = 0.044
*wR*(*F* ^2^) = 0.106
*S* = 1.05
1592 reflections
198 parameters
1 restraint
H atoms treated by a mixture of independent and constrained refinement
Δρ_max_ = 0.19 e Å^−3^
Δρ_min_ = −0.18 e Å^−3^
Absolute structure: 1004 measured Friedel pairs merged for refinement

### 

Data collection: *CrysAlis CCD* (Oxford Diffraction, 2009 ▶); cell refinement: *CrysAlis RED* (Oxford Diffraction, 2009 ▶); data reduction: *CrysAlis RED*; program(s) used to solve structure: *SHELXS97* (Sheldrick, 2008 ▶); program(s) used to refine structure: *SHELXL97* (Sheldrick, 2008 ▶); molecular graphics: *SHELXTL* (Sheldrick, 2008 ▶) and Mercury (Macrae *et al.*, 2008 ▶); software used to prepare material for publication: *SHELXTL*.

## Supplementary Material

Crystal structure: contains datablock(s) I, global. DOI: 10.1107/S1600536812039359/mw2082sup1.cif

Structure factors: contains datablock(s) I. DOI: 10.1107/S1600536812039359/mw2082Isup2.hkl

Supplementary material file. DOI: 10.1107/S1600536812039359/mw2082Isup3.cml

Additional supplementary materials:  crystallographic information; 3D view; checkCIF report

## Figures and Tables

**Table 1:** Hydrogen-bond geometry (Å, °)

*D*—H⋯*A*	*D*—H	H⋯*A*	*D*⋯*A*	*D*—H⋯*A*
O3—H3⋯O2^i^	0.92 (5)	1.84 (5)	2.752 (4)	169 (5)
O2—H2⋯O1^ii^	0.90 (4)	1.89 (4)	2.723 (3)	154 (3)

Symmetry codes: (i) 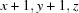; (ii) 
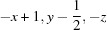
.

**Fig. 1.**
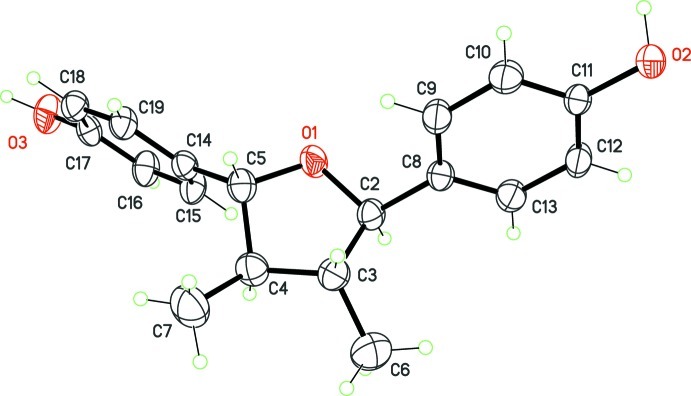
ORTEP-like view of the title molecule with displacement ellipsoids for non-H atoms at the 30% probability level.

**Fig. 2.**
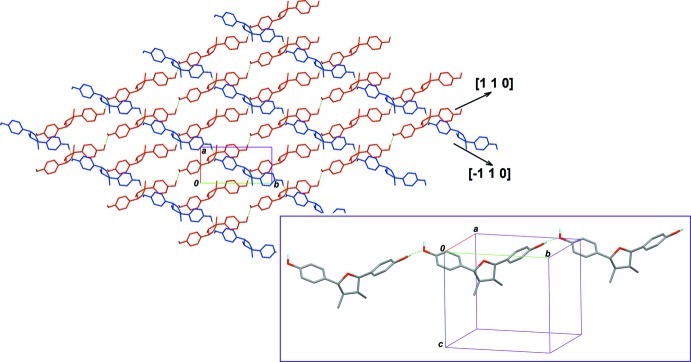
Part of the crystal structure of the title compound viewed down c emphasizing the chain framework. All red chains are placed in a common plane, and blue chains are in a plane above the red molecules. Both planes interact through O—H···O(furan) contacts. The inset represents a part of a single chain. All H atoms not involved in hydrogen bonds have been omitted, and intermolecular contacts are represented as green dashed lines.

## supplementary crystallographic information

### Comment

The characterization of the title molecule is a part of a long-term project related to the screening of extracts obtained from plants used in Mexican traditional medicine to treat respiratory infections like tuberculosis. The title furanoid lignan is present in the chloroformic extract of *Larrea tridentata*, a plant found mainly in the southwestern US and northern Mexico. We have recently probed the antibacterial and antimycobacterial activity of this molecule and found that it is active against methicillin resistant *S. aureus* and *M. tuberculosis* H37Rv strain (Favela-Hernández *et al.*, 2012). This molecule was previously extracted from *L. tridentata* samples from Phoenix, Arizona and characterized by MS and NMR (Konno *et al.*, 1990). The synthesis and chiral HPLC analysis of stereoisomers of this compound have also been carried out (Moinuddin *et al.*, 2003).

The relative stereochemistry for the four chiral C atoms in the furan ring was determined (Fig. 1) showing that the crystallized lignan corresponds to 4-*epi*-larreatricin (Konno *et al.*, 1990; Moinuddin *et al.*, 2003). The same configuration was found in related furanoid lignans from other natural sources, for example grandisin, which is extracted from *Cryptocarya crassinervia* (Soepadamo *et al.*, 1991). The four substituents of the central furan ring are arranged in an all-*trans*α,α'-diaryl-β,β'-dimethyl manner thus avoiding steric hindrance between aryl and methyl groups. The furan ring adopts a twisted envelope conformation characteristic of ribose sugars in the B-form of DNA (hydrated DNA). This may be checked by computing the phase angle of pseudorotation for the ring, *P* = 171.3°, and the maximum degree of pucker, ν_m_ = 37.7° (Altona & Sundaralingam, 1972). The comparison of these data with the distribution of *P* and ν_m_ for β-nucleosides found in the CSD clearly shows that the title compound lies in the south hemisphere of the pseudorotational wheel and within the C2'-*endo* cluster (^2^*E* form, *P* = 162° (See Fig. 3 in Altona & Sundaralingam, 1972 and Fig. 6 in Sun *et al.*, 2004)).

The crystal structure is based on chains formed through intermolecular O—H···O hydrogen bonds involving the hydroxyl groups (Fig. 2, inset). The resulting layer interacts with the neighboring layer packed along the *c* axis, through O—H···O(furan) contacts, forming the complete three-dimensional framework (Fig. 2).

### Experimental

Leaves of *L. tridentata* were collected in Galeana, Nuevo León, Mexico, and authenticated by Biól. Mauricio González (Voucher 024772, Facultad de Ciencias Biológicas, UANL). Dried and ground leaves (500 g) were extracted with hexane and then with CHCl_3_ through maceration. Details of the chromatography of the chloroform fraction have been described previously (Favela-Hernández *et al.*, 2012). This afforded, among other products, 11 mg of the title molecule, which was crystallized from CHCl_3_/MeOH (95/5, v/v). m.p. 503–505 K (Lit. 503–505 K, Konno *et al.*, 1990). ^1^H and ^13^C NMR data are in agreement with the X-ray structure.

### Refinement

With only first row elements present, the absolute structure could not be determined with certainty so the Friedel pairs (1004) were merged. C-bound H atoms were placed in idealized positions with C—H = 0.93 (aromatic CH), 0.96 (methyl CH_3_) or 0.98 Å (methine CH) and included as riding contributions. Hydroxyl H atoms, H2 and H3, were found in a difference map and refined freely. Isotropic displacement parameters for H atoms were calculated as *U*_iso_(H) = *xU*_eq_(carrier atom) where *x* = 1.5 for methyl and hydroxyl groups, and *x* = 1.2 for other H atoms.

### Crystal data

**Table:**

C_18_H_20_O_3_	*F*(000) = 304
*M**_r_* = 284.34	*D*_x_ = 1.222 Mg m^−^^3^
Monoclinic, *P*2_1_	Melting point: 503 K
Hall symbol: P 2yb	Mo *K*α radiation, λ = 0.71073 Å
*a* = 6.4225 (4) Å	Cell parameters from 1308 reflections
*b* = 12.4973 (7) Å	θ = 3.5–26.0°
*c* = 9.8176 (7) Å	µ = 0.08 mm^−^^1^
β = 101.243 (6)°	*T* = 298 K
*V* = 772.88 (9) Å^3^	Block, colourless
*Z* = 2	0.36 × 0.26 × 0.21 mm

### Data collection

**Table:**

Agilent Xcalibur (Atlas, Gemini) diffractometer	1592 independent reflections
Radiation source: Enhance (Mo) X-ray Source	1107 reflections with *I* > 2σ(*I*)
Graphite monochromator	*R*_int_ = 0.041
Detector resolution: 10.4685 pixels mm^-1^	θ_max_ = 26.1°, θ_min_ = 3.5°
φ and ω scans	*h* = −7→6
Absorption correction: analytical [*CrysAlis PRO* (Oxford Diffraction, 2009), based on expressions derived by Clark & Reid (1995)]	*k* = −15→15
*T*_min_ = 0.980, *T*_max_ = 0.985	*l* = −12→11
5223 measured reflections	

### Refinement

**Table:**

Refinement on *F*^2^	Primary atom site location: structure-invariant direct methods
Least-squares matrix: full	Secondary atom site location: difference Fourier map
*R*[*F*^2^ > 2σ(*F*^2^)] = 0.044	Hydrogen site location: inferred from neighbouring sites
*wR*(*F*^2^) = 0.106	H atoms treated by a mixture of independent and constrained refinement
*S* = 1.05	*w* = 1/[σ^2^(*F*_o_^2^) + (0.0498*P*)^2^] where *P* = (*F*_o_^2^ + 2*F*_c_^2^)/3
1592 reflections	(Δ/σ)_max_ < 0.001
198 parameters	Δρ_max_ = 0.19 e Å^−^^3^
1 restraint	Δρ_min_ = −0.18 e Å^−^^3^
0 constraints	Absolute structure: 1004 measured Friedel pairs merged for refinement

### Fractional atomic coordinates and isotropic or equivalent isotropic displacement parameters (Å^2^)

**Table:**

	*x*	*y*	*z*	*U*_iso_*/*U*_eq_	
O1	0.6576 (4)	0.17906 (19)	0.2077 (2)	0.0608 (7)	
O2	0.3301 (4)	−0.29301 (19)	0.0656 (2)	0.0569 (6)	
H2	0.377 (5)	−0.303 (4)	−0.014 (4)	0.085*	
O3	0.9213 (4)	0.64265 (18)	0.0690 (3)	0.0741 (8)	
H3	1.056 (8)	0.659 (4)	0.058 (5)	0.111*	
C2	0.5625 (5)	0.1142 (3)	0.3000 (3)	0.0489 (8)	
H2A	0.4333	0.1500	0.3155	0.059*	
C3	0.7244 (5)	0.1130 (3)	0.4373 (4)	0.0588 (9)	
H3A	0.8255	0.0553	0.4313	0.071*	
C4	0.8399 (5)	0.2166 (3)	0.4337 (3)	0.0598 (9)	
H4A	0.7472	0.2739	0.4551	0.072*	
C5	0.8532 (5)	0.2269 (3)	0.2820 (4)	0.0519 (9)	
H5A	0.9727	0.1835	0.2651	0.062*	
C6	0.6305 (7)	0.0920 (5)	0.5619 (4)	0.0953 (16)	
H6A	0.7419	0.0872	0.6426	0.143*	
H6B	0.5530	0.0259	0.5498	0.143*	
H6C	0.5362	0.1493	0.5737	0.143*	
C7	1.0528 (6)	0.2277 (4)	0.5332 (4)	0.0903 (14)	
H7A	1.1126	0.2967	0.5212	0.135*	
H7B	1.1476	0.1728	0.5140	0.135*	
H7C	1.0323	0.2207	0.6270	0.135*	
C8	0.5031 (5)	0.0063 (2)	0.2373 (4)	0.0459 (8)	
C9	0.6388 (5)	−0.0506 (3)	0.1717 (4)	0.0564 (9)	
H9A	0.7700	−0.0214	0.1659	0.068*	
C10	0.5841 (5)	−0.1507 (3)	0.1138 (4)	0.0574 (9)	
H10A	0.6780	−0.1878	0.0700	0.069*	
C11	0.3913 (5)	−0.1941 (3)	0.1217 (3)	0.0448 (8)	
C12	0.2553 (5)	−0.1400 (3)	0.1891 (4)	0.0561 (9)	
H12A	0.1254	−0.1701	0.1963	0.067*	
C13	0.3120 (5)	−0.0407 (3)	0.2462 (4)	0.0571 (9)	
H13A	0.2189	−0.0047	0.2918	0.069*	
C14	0.8752 (5)	0.3372 (3)	0.2270 (3)	0.0467 (8)	
C15	0.7158 (5)	0.4127 (3)	0.2230 (4)	0.0586 (10)	
H15A	0.5943	0.3948	0.2563	0.070*	
C16	0.7343 (5)	0.5140 (3)	0.1703 (4)	0.0615 (10)	
H16A	0.6250	0.5632	0.1675	0.074*	
C17	0.9120 (5)	0.5420 (3)	0.1225 (4)	0.0526 (9)	
C18	1.0742 (5)	0.4688 (3)	0.1258 (4)	0.0535 (9)	
H18A	1.1962	0.4877	0.0937	0.064*	
C19	1.0533 (5)	0.3675 (3)	0.1771 (4)	0.0516 (9)	
H19A	1.1621	0.3182	0.1781	0.062*	

### Atomic displacement parameters (Å^2^)

**Table:**

	*U*^11^	*U*^22^	*U*^33^	*U*^12^	*U*^13^	*U*^23^
O1	0.0838 (15)	0.0455 (15)	0.0496 (14)	−0.0226 (12)	0.0047 (11)	0.0036 (11)
O2	0.0778 (15)	0.0387 (14)	0.0569 (15)	−0.0091 (12)	0.0199 (11)	−0.0006 (13)
O3	0.0794 (17)	0.0361 (15)	0.109 (2)	−0.0074 (13)	0.0236 (16)	0.0085 (14)
C2	0.0552 (18)	0.037 (2)	0.055 (2)	−0.0024 (15)	0.0118 (15)	0.0019 (16)
C3	0.070 (2)	0.051 (2)	0.055 (2)	−0.0058 (18)	0.0119 (17)	0.0035 (19)
C4	0.063 (2)	0.059 (2)	0.054 (2)	−0.0017 (18)	0.0007 (15)	0.006 (2)
C5	0.0527 (19)	0.039 (2)	0.063 (2)	−0.0002 (15)	0.0098 (15)	−0.0003 (18)
C6	0.107 (3)	0.109 (4)	0.069 (3)	−0.019 (3)	0.013 (2)	0.015 (3)
C7	0.086 (3)	0.097 (4)	0.077 (3)	−0.015 (3)	−0.009 (2)	0.002 (3)
C8	0.0547 (19)	0.0343 (19)	0.049 (2)	−0.0024 (14)	0.0109 (15)	0.0031 (15)
C9	0.0549 (19)	0.048 (2)	0.070 (3)	−0.0090 (16)	0.0216 (17)	−0.006 (2)
C10	0.058 (2)	0.053 (2)	0.067 (2)	0.0024 (17)	0.0249 (17)	−0.005 (2)
C11	0.0588 (19)	0.0306 (18)	0.0461 (19)	−0.0029 (15)	0.0128 (14)	0.0048 (15)
C12	0.0563 (19)	0.043 (2)	0.073 (3)	−0.0117 (17)	0.0253 (17)	−0.0023 (19)
C13	0.062 (2)	0.048 (2)	0.067 (3)	−0.0035 (17)	0.0257 (17)	−0.0069 (19)
C14	0.0513 (18)	0.038 (2)	0.050 (2)	−0.0052 (14)	0.0076 (14)	−0.0030 (16)
C15	0.054 (2)	0.043 (2)	0.081 (3)	−0.0055 (16)	0.0191 (18)	−0.0015 (19)
C16	0.057 (2)	0.040 (2)	0.088 (3)	−0.0006 (16)	0.0154 (18)	0.003 (2)
C17	0.060 (2)	0.0313 (19)	0.064 (2)	−0.0072 (16)	0.0073 (16)	−0.0056 (17)
C18	0.0538 (19)	0.045 (2)	0.064 (2)	−0.0055 (16)	0.0176 (16)	−0.0077 (18)
C19	0.0507 (18)	0.037 (2)	0.068 (2)	0.0000 (15)	0.0155 (16)	−0.0045 (17)

### Geometric parameters (Å, º)

**Table:**

O1—C2	1.437 (4)	C7—H7C	0.9600
O1—C5	1.452 (3)	C8—C9	1.378 (5)
O2—C11	1.378 (4)	C8—C13	1.379 (4)
O2—H2	0.90 (4)	C9—C10	1.390 (5)
O3—C17	1.368 (4)	C9—H9A	0.9300
O3—H3	0.92 (5)	C10—C11	1.368 (4)
C2—C8	1.501 (4)	C10—H10A	0.9300
C2—C3	1.533 (5)	C11—C12	1.373 (5)
C2—H2A	0.9800	C12—C13	1.381 (5)
C3—C6	1.489 (5)	C12—H12A	0.9300
C3—C4	1.496 (5)	C13—H13A	0.9300
C3—H3A	0.9800	C14—C19	1.383 (5)
C4—C5	1.513 (5)	C14—C15	1.387 (4)
C4—C7	1.524 (4)	C15—C16	1.381 (5)
C4—H4A	0.9800	C15—H15A	0.9300
C5—C14	1.497 (5)	C16—C17	1.362 (5)
C5—H5A	0.9800	C16—H16A	0.9300
C6—H6A	0.9600	C17—C18	1.382 (5)
C6—H6B	0.9600	C18—C19	1.379 (5)
C6—H6C	0.9600	C18—H18A	0.9300
C7—H7A	0.9600	C19—H19A	0.9300
C7—H7B	0.9600		
			
C2—O1—C5	110.3 (2)	H7B—C7—H7C	109.5
C11—O2—H2	111 (3)	C9—C8—C13	117.5 (3)
C17—O3—H3	112 (3)	C9—C8—C2	121.5 (3)
O1—C2—C8	110.7 (3)	C13—C8—C2	121.0 (3)
O1—C2—C3	105.1 (2)	C8—C9—C10	121.5 (3)
C8—C2—C3	115.2 (3)	C8—C9—H9A	119.2
O1—C2—H2A	108.5	C10—C9—H9A	119.2
C8—C2—H2A	108.5	C11—C10—C9	119.6 (3)
C3—C2—H2A	108.5	C11—C10—H10A	120.2
C6—C3—C4	117.0 (4)	C9—C10—H10A	120.2
C6—C3—C2	114.2 (3)	C10—C11—C12	120.0 (3)
C4—C3—C2	103.0 (3)	C10—C11—O2	121.6 (3)
C6—C3—H3A	107.4	C12—C11—O2	118.4 (3)
C4—C3—H3A	107.4	C11—C12—C13	119.7 (3)
C2—C3—H3A	107.4	C11—C12—H12A	120.1
C3—C4—C5	102.7 (3)	C13—C12—H12A	120.1
C3—C4—C7	116.8 (3)	C8—C13—C12	121.7 (3)
C5—C4—C7	114.0 (3)	C8—C13—H13A	119.2
C3—C4—H4A	107.6	C12—C13—H13A	119.2
C5—C4—H4A	107.6	C19—C14—C15	117.4 (3)
C7—C4—H4A	107.6	C19—C14—C5	121.5 (3)
O1—C5—C14	109.3 (2)	C15—C14—C5	121.1 (3)
O1—C5—C4	104.5 (2)	C16—C15—C14	121.1 (3)
C14—C5—C4	117.4 (3)	C16—C15—H15A	119.4
O1—C5—H5A	108.4	C14—C15—H15A	119.4
C14—C5—H5A	108.4	C17—C16—C15	120.3 (3)
C4—C5—H5A	108.4	C17—C16—H16A	119.9
C3—C6—H6A	109.5	C15—C16—H16A	119.9
C3—C6—H6B	109.5	C16—C17—O3	118.1 (3)
H6A—C6—H6B	109.5	C16—C17—C18	120.0 (3)
C3—C6—H6C	109.5	O3—C17—C18	121.9 (3)
H6A—C6—H6C	109.5	C19—C18—C17	119.3 (3)
H6B—C6—H6C	109.5	C19—C18—H18A	120.3
C4—C7—H7A	109.5	C17—C18—H18A	120.3
C4—C7—H7B	109.5	C18—C19—C14	121.8 (3)
H7A—C7—H7B	109.5	C18—C19—H19A	119.1
C4—C7—H7C	109.5	C14—C19—H19A	119.1
H7A—C7—H7C	109.5		
			
C5—O1—C2—C8	−131.3 (3)	C8—C9—C10—C11	0.1 (5)
C5—O1—C2—C3	−6.2 (3)	C9—C10—C11—C12	−1.4 (5)
O1—C2—C3—C6	155.4 (4)	C9—C10—C11—O2	179.9 (3)
C8—C2—C3—C6	−82.5 (4)	C10—C11—C12—C13	1.4 (5)
O1—C2—C3—C4	27.4 (3)	O2—C11—C12—C13	−179.9 (3)
C8—C2—C3—C4	149.6 (3)	C9—C8—C13—C12	−1.4 (5)
C6—C3—C4—C5	−163.5 (3)	C2—C8—C13—C12	−179.9 (3)
C2—C3—C4—C5	−37.3 (3)	C11—C12—C13—C8	0.1 (5)
C6—C3—C4—C7	71.0 (5)	O1—C5—C14—C19	−124.5 (3)
C2—C3—C4—C7	−162.8 (3)	C4—C5—C14—C19	116.8 (3)
C2—O1—C5—C14	−143.7 (3)	O1—C5—C14—C15	54.8 (4)
C2—O1—C5—C4	−17.2 (3)	C4—C5—C14—C15	−63.9 (4)
C3—C4—C5—O1	34.0 (3)	C19—C14—C15—C16	0.3 (5)
C7—C4—C5—O1	161.3 (3)	C5—C14—C15—C16	−179.0 (3)
C3—C4—C5—C14	155.2 (3)	C14—C15—C16—C17	−0.7 (6)
C7—C4—C5—C14	−77.5 (4)	C15—C16—C17—O3	178.8 (3)
O1—C2—C8—C9	44.0 (4)	C15—C16—C17—C18	0.4 (6)
C3—C2—C8—C9	−75.1 (4)	C16—C17—C18—C19	0.3 (5)
O1—C2—C8—C13	−137.5 (3)	O3—C17—C18—C19	−178.0 (3)
C3—C2—C8—C13	103.4 (4)	C17—C18—C19—C14	−0.7 (5)
C13—C8—C9—C10	1.3 (5)	C15—C14—C19—C18	0.4 (5)
C2—C8—C9—C10	179.9 (3)	C5—C14—C19—C18	179.7 (3)

### Hydrogen-bond geometry (Å, º)

**Table:**

*D*—H···*A*	*D*—H	H···*A*	*D*···*A*	*D*—H···*A*
O3—H3···O2^i^	0.92 (5)	1.84 (5)	2.752 (4)	169 (5)
O2—H2···O1^ii^	0.90 (4)	1.89 (4)	2.723 (3)	154 (3)

Symmetry codes: (i) *x*+1, *y*+1, *z*; (ii) −*x*+1, *y*−1/2, −*z*.
